# The prevalence and determinants of catastrophic health expenditures attributable to non-communicable diseases in low- and middle-income countries: a methodological commentary

**DOI:** 10.1186/s12939-014-0107-1

**Published:** 2014-11-07

**Authors:** Yevgeniy Goryakin, Marc Suhrcke

**Affiliations:** Norwich Medical School, University of East Anglia, Norwich, NR4 7TJ UK; UKCRC Centre for Diet and Activity Research (CEDAR), Institute of Public Health, Cambridge, UK; Centre for Health Economics, University of York, York, UK

**Keywords:** Catastrophic out-of-pocket expenditures, Non-communicable diseases, Developing countries

## Abstract

**Background:**

Non-communicable diseases (NCDs), while traditionally considered a “rich world”-problem, have been spreading fast in low and middle income countries and by now account for a large share of mortality and ill-health in these countries, too. In addition to the disease burden, NCDs may also impose a substantial economic cost. One way in which NCDs might impact people’s economic well-being may be via the out-of-pocket expenditures required to cover treatment and other costs associated with suffering from an NCD.

**Methods:**

In this commentary, we identify and discuss the methodological challenges related to cross-country comparison of-out-of-pocket and catastrophic out-of-pocket health care expenditures, attributable to NCDs, focussing on low and middle income countries.

**Results:**

There is significant evidence of substantial cost burden placed by NCDs on patients living in low and middle income countries, with most of it being heavily concentrated among low socioeconomic status groups. However, a large variation in definition of COOPE between studies prevents cross-country comparison. In addition, as most studies tend to be observational, causal inferences are often not possible. This is further complicated by the cross-sectional nature of studies, small sample sizes, and/or limited duration of follow-up of patients. Most evidence for certain conditions (e.g., cancer) tends to be collected in high-income countries only.

**Conclusions:**

The definitions for COOPEs should be standardized as much as possible, to enable comparison of COOPE prevalence between countries. Prospective study design using larger samples representative of broader sections of local population, collecting better data on both direct and indirect treatment costs is also needed.

**Electronic supplementary material:**

The online version of this article (doi:10.1186/s12939-014-0107-1) contains supplementary material, which is available to authorized users.

## Introduction

Non-communicable diseases (NCDs), while traditionally considered a “rich world”-problem, have been spreading fast in low and middle income countries (LMICs) and by now account for a large share of mortality and ill-health in these countries, too [1,2]. In addition to the disease burden, NCDs may also impose a substantial economic cost. This is particularly likely in LMICs that lack appropriate health spending pre-payment systems, such as health insurance [3]. When out-of-pocket medical care expenditures are very large, relative to the material resources available to the household, they may become “catastrophic” [4].

In this paper we identify and discuss a series of methodological challenges related to cross-country comparison of out-of-pocket expenditures (OOPE) and catastrophic out-of-pocket health care expenditures (COOPEs), attributable to NCDs, focussing on LMICs. These challenges have been identified on the basis of an extensive review of the relevant literature, the details of which are available in the Additional files 1 and 2.

### Variable definition

Cross-country comparison of COOPE prevalence is greatly complicated by the fact that the definition of COOPEs varies between studies [5], although in at least one study, prevalence was estimated in multiple countries using a common definition [6].

The two main components in estimating COOPEs are household-specific OOPEs, which enters the numerator, and some measure of household resources, i.e. typically income, consumption or spending [7], which enters the denominator. The simplest approach is to use total income in the denominator [8,9]. The problem with this strategy is that it may lead to the underestimation of the extent of COOPEs, as it does not take into account other basic needs (e.g. food). Therefore, one widely used alternative approach is to define COOPEs as total out-of-pocket expenditures on health as a proportion of total expenditures or income, net of some commonly-defined poverty line measure of resources, such as one dollar per day income or spending [5]. However, as it does not take into account location-specific costs to satisfy basic needs, it may give a distorted picture of COOPEs prevalence in any given country. To account for this, the denominator may be alternatively defined as income or expenditures net of essential spending, such as on food [5,7]. However, the drawback of this approach is that it may compromise cross-country comparisons, as the cost of essential items may vary a great deal across locations.

### Thresholds

Another complication is that COOPEs are often defined in relation to a specific threshold (e.g., health expenditures accounting for at least 20% of disposable income). However, such thresholds often vary between studies, and therefore cross-country comparisons may be difficult when non-standard thresholds are used. In practice, lower thresholds (e.g., 10%) are typically used to define COOPEs when total expenditures are in the denominator, while considerably higher ones (e.g. 40%) are applied when non-food expenditures are part of the denominator [7].

### Income vs consumption

As the main purpose of estimating COOPE prevalence is to study the effect of illness on living standards, using income instead of consumption (e.g., proxied by expenditures) in the denominator may not necessarily address this purpose [7]. For example, the evidence suggests that some households may be able to finance high OOPEs on health in a number of ways, e.g. by selling their assets or utilizing their savings [6,10-12], by increasing their income transfers [11], by accepting help from their extended family or friends [6], or by some other measures such as borrowing and access to microfinance [10,12-15]. This may not be obvious if the denominator is defined by income rather than spending [7].

### Definition of OOPE

An additional complication is that the definition of out-of-pocket expenditures may vary between studies, and may [8,16-18] or may not [9,19,20] include spending on indirect items such as cost of travel, employment-related losses, or caretaker burden. As those cost elements are usually not covered by health insurance, they may need to be considered as part of OOPEs. Thus, some reviewed studies found that earnings losses may be significantly larger than medical OOPEs [7,18]. In Sri Lanka, it was found that although public health system use is free at the point of delivery and OOPEs on health care are modest [21], sometimes several direct and indirect costs either prevent people from following diabetes treatment regimens as prescribed, or expose them to greater risk of economic hardship [16].

Even the definitions employed for direct costs vary substantially between studies, with some including expenditures on tests and diagnostics and others not. In addition, assumptions are sometimes made about the costs of drugs and the patterns of their use, or on income level or frequency of disease occurrence rather than on actual records [22,23]. Also, most metrics designed to estimate prevalence of COOPE do not take into account households that may choose to forego medical treatment altogether because it may be too expensive for them [7,24]. An alternative definition to address this specific complication is to measure exposure to, rather than the actual payment of OOPE [7], although accurate collection of such data may be very challenging.

### Sampling issues

There is considerable heterogeneity between studies in sample sizes, ranging from very small (e.g., 32 families living in one country [8], or 34 patients with chronic conditions- in another [25]), to much larger ones (121,051 individuals living in 35 countries [26], or 39,060 people living in one country [9]). In some studies, sampling was done on the household level [11,27], while in others (e.g., [17]) on the individual level.

Another serious sampling problem relates to self-selection, or to sampling from unrepresentative groups. While in some reviewed studies the samples are nationally representative [9], this is not the case in many other studies. For example, the selected sample is often restricted to hospitalized patients only (e.g., [6,28]), or to children [8]. In other cases, some population groups may be deliberately oversampled (e.g., wealthier households [29]), which may complicate generalizing to other population groups [30]. If, for instance, hospitalized patients are in worse health than the population at large, then the population-level probability of incurring COOPE may be overestimated. On the other hand, the cost estimates for such patients may be under-estimated if their costs in the community are not followed up upon their discharge from hospital. Likewise, if hospitalization costs are very high in some countries, and hence only wealthier (and relatively healthier) people are likely to be admitted, then again such results may be difficult to compare with studies where such problems do not exist. In addition there may be reporting bias, as patients tend to have difficulty keeping track of their income/expenditure for an extended period of time [6].

### Follow-up issues

Follow-up times do vary between studies and are commonly rather short (e.g. up to 12 months), potentially limiting cross-country comparability as well as capturing the long-term effect of NCDs [31]. Some conditions (e.g. stroke) may require extended and costly rehabilitation periods in the community [31], which are rarely followed up in studies. LMICs in particular tend to lack longitudinal data of sufficient duration [32], although some studies have used panel datasets [11,15], or at least repeated cross-sectional design [33]. Expenditures on some diseases (eg cancer) may also exhibit nonlinear patterns. For example, health care expenditures tend to be the highest in the first 6 months after diagnosis, and 12 months before death [34]. Therefore, the estimated average costs may to a large extent depend on the overlap of the follow-up period with the course of the disease. Similarly, if average rather than actual costs of drugs are analyzed, estimated OOPEs may be misleading, especially if drug prices fluctuate considerably.

### Research design

A majority of studies simply report descriptive statistics on how the risk of incurring COOPE varies by different patient characteristics. Some studies assess the average total cost of care among patients with a specified chronic condition [19,22], generally in relation to the annual or monthly income measure [17], while some others provide mean values of OOPE/COOPE, without restricting the sample to chronic patients [10], or without any comparison in these outcomes between patients with and without chronic illnesses [16]. This may lead to an overestimate of the NCD-attributable expenditures, although some studies do have a reference group, or at least a random population sample [35] for comparison.

To account for the possibility that OOPEs may differ between patients for reasons other than having a chronic illness, some studies examine the correlation between having a chronic disease and OOPE while also controlling for several potential (mostly socioeconomic, demographic and health) confounders [11,26,32]. However, this is not standard practice, which makes cross-country-comparison of results more difficult.

Overall, the cross-sectional design is most frequently employed in this literature – a feature that tends to limit causal inference, as important unobservable determinants may be located at the individual level, which country fixed effects and existing controls cannot account for [26].

Although one author did take advantage of the longitudinal nature of the data and employed instrumental variable estimators to control for potential endogeneity of chronic disease indicators [11], the choice of the instruments did not appear to be fully justified.

### Other issues regarding cross-country comparisons

A number of other issues have also been found to potentially affect cross-country comparisons of prevalence and determinants of NCD-related COOPE. Thus, in many cases, self-reported information on diseases and conditions is used, rather than actual diagnosis [11,26,32,36]. For example, measured information on fasting blood glucose is often unavailable, and the diagnosis is therefore self-reported, although usually based on prior diagnosis (which may depend on access to health services). Similarly, the use of self-reported medical spending may be subject to recall bias, or to over- or under-reporting related to socioeconomic status (SES).

The patient mix is also often difficult to compare. For example, in some studies, patients with both chronic and non-chronic disease have been included, without a clear distinction [17,29]. Similarly, some authors (e.g. [13,14]) looked at the effect of changes in ability to perform various activities of daily living (ADL) on medical spending, which they argue are important proxies for physical functioning ability. However, it is not clear to what extent such changes are due to NCDs rather than communicable or other conditions.

Estimating prevalence of COOPE is further complicated by the fact that in some countries, average OOPEs may be relatively low not because the treatments are cheap, but because they are estimated conditional on receiving treatment. If they are perceived as unaffordable by a large proportion of the population, and these costs are therefore not incurred [6,8,36], an erroneous conclusion may be drawn that COOPE prevalence is low.

We also found that the strength of the relationship between having a disease and incurring COOPE may vary not only by disease, but also by national income level [26], highlighting the danger of extrapolating findings between countries at different levels of economic development. The magnitude (as opposed to prevalence) of COOPE may also differ depending on the national level of income, which is difficult to capture if a threshold-based measure of COOPEs (e.g., 30% of expenditures accounted for by OOPE) is used. For example, in one of the poorest countries in the world – Tajikistan – one episode of hospital stay could cost as much as patients’ total annual income [10], with about 70% of the poorest quintile reporting that patients did not buy drugs even though they had a medical prescription, because they could not afford to.

Finally, some studies have attempted to measure SES-related inequalities in the prevalence of COOPE [6,8,19,31,36-40], and generally found that those with the least income/education, or living in the poorest countries are most likely to incur large OOPE [6,8,19,31,36,37], and that when health care is consumed, financial burden appears to concentrate heavily among patients in lower SES status [9,10,18,20,23,33,36,41]. Nevertheless, despite the existence of standard approaches for measuring COOPE-related inequalities [7], including indirect and direct standardization methods, very few if any studies utilize them.

## Conclusions

In this paper we have provided – based on an in depth review of the relevant evidence base – a discussion of the methodological challenges facing researchers who are interested in comparing prevalence and determinants of NCD-related COOPEs across countries.

It is important to emphasize that while another recent systematic review [42] considered financial burden from non-communicable diseases in LMICs, our commentary is different in two important respects. First, we are much more focussed on COOPE (as opposed to “financial burden”) than Kankeu et al [42]. Second, we dedicate a particularly significant share of the discussion on methodological issues and challenges in these studies.

It is also important to be mindful of the limitations of our discussion, and in particular of the underlying review. Thus, we cannot exclude the possibility that we may have overlooked some relevant evidence, especially in the non-English literature. Furthermore, although we tried to review as broad a selection of articles as possible, our review was not truly systematic, and may therefore have overlooked some relevant articles. Nevertheless, the sample of studies that we did find may be expected to cover the predominant part of the relevant body of work in this field, and our discussion suggests a number of lessons for future research:

First, if the goal is to facilitate comparisons of COOPE prevalence between countries, the definitions for both numerator and denominator, as well as the threshold level at which OOPEs become catastrophic, should be standardized as much as possible. The comparisons should ideally be based on the common length of the follow-up period, as well as on common disease definitions, preferably determined by clinical assessment.

Second, our study has confirmed the existence of a significant socioeconomic gradient in the prevalence of chronic disease-related COOPE in developing countries. Given the important equity implications of this finding, greater effort should be directed at more uniform measurement of such gradient across countries. For example, the direct or indirect standardization for age and gender and the use of concentration indices, may facilitate cross-country comparisons of socioeconomic inequalities across countries.

Third, comparisons will also be facilitated by using similar sampling frames (preferably not restricted to hospitalized patients only), and appropriate designs to elicit sufficient response rates.

Fourth, to evaluate the true extent of COOPE, better data on indirect costs should be collected. This is especially true for countries with more generous health insurance systems, where direct costs may be covered well, as well as for very low-income countries, where cost components such as travel costs may be prohibitive.

Fifth, more reliable data on medication costs and their use should be collected in LMICs, given their importance in OOPE.

Sixth, prospective data collection is preferred to avoid recall bias problems. In addition, real rather than estimated or assumed cost/utilisation data should be used.

Finally, more studies should be conducted to address the potential problems of the endogeneity of chronic illness.

## Additional files

Additional file 1:
**Search strategy and outcomes.**
Additional file 2:
**Prisma flowchart for the literature search strategy.**
