# Supplementary material for: The prevalence and determinants of catastrophic health expenditures attributable to non-communicable diseases in low- and middle-income countries: a methodological commentary
Source: Int J Equity Health. 2014 Nov 7;13:107. doi: 10.1186/s12939-014-0107-1 (PMC4228103; doi:10.1186/s12939-014-0107-1)
Supplement: Additional file 1: — Search strategy and outcomes. [file 12939_2014_107_MOESM1_ESM.docx]

**Additional file 1: Search strategy and outcomes**

1. Search in Econlit, using the following terms:

- (chronic OR diabetes OR cancer OR cardiovascular OR respiratory) AND ( poverty OR expenditure OR spending OR catastrophic OR out of pocket ), limited to articles from 1990 until 2013, and to those published in peer-reviewed journals: 408 abstracts found

2. Search in Pubmed had to be necessarily restricted to the last 10 years, due to a very large number of search results. We used the following terms:

- (chronic[All Fields] OR "diabetes mellitus"[MeSH Terms] OR "neoplasms"[MeSH Terms] OR "cardiovascular system"[MeSH Terms] OR "cardiovascular"[All Fields] OR "respiratory tract diseases"[MeSH Terms]) AND "health expenditures"[MeSH Terms] AND ("2003/01/01"[PDAT] : "2012/12/31"[PDAT]). We ended up with 1,057 potentially useful abstracts

4. In addition, we screened references from two recent reviews, looking for potentially relevant articles not selected in steps 1 and 2. The first one explored the link between NCDs and poverty in low and middle income countries ([Niessen LW, 2013](#_ENREF_2)). We also considered the recent review by ([Kankeu et al., 2013](#_ENREF_1)) already mentioned above.

5. In the first stage, we selected the relevant abstracts, after which we considered specific articles in this group. Out of shortlisted 387 articles, we selected our core group of papers on the basis of their potential relevance to the research question. The exclusion criteria were based on whether abstracts and articles dealt with our research question of interest, and whether they applied to low and middle income countries.

**Figure 1. Prisma flowchart for the literature search strategy**
