# Supplementary material for: The prevalence and determinants of catastrophic health expenditures attributable to non-communicable diseases in low- and middle-income countries: a methodological commentary
Source: Int J Equity Health. 2014 Nov 7;13:107. doi: 10.1186/s12939-014-0107-1 (PMC4228103; doi:10.1186/s12939-014-0107-1)
Supplement: Additional file 2: — Prisma flowchart for the literature search strategy. [file 12939_2014_107_MOESM2_ESM.docx]

**Additional file 2. Prisma flowchart for the literature search strategy**

Full-text articles selected
(n = 42)

Additional articles identified through references in selected articles

(n= 5)

Abstracts excluded
(n = 1,093)

Additional records identified through Pubmed
(n= 1,057)

Records identified through Econlit search (n= 408 )

Articles selected for further screening (n (n= 387)

Abstracts screened
(n =1,480 )

Full-text articles excluded
(n = 350 )
